# Prenatal Metformin Exposure in Mice Programs the Metabolic Phenotype of the Offspring during a High Fat Diet at Adulthood

**DOI:** 10.1371/journal.pone.0056594

**Published:** 2013-02-15

**Authors:** Henriikka Salomäki, Laura H. Vähätalo, Kirsti Laurila, Norma T. Jäppinen, Anna-Maija Penttinen, Liisa Ailanen, Juan Ilyasizadeh, Ullamari Pesonen, Markku Koulu

**Affiliations:** 1 Institute of Biomedicine, Department of Pharmacology, Drug Development and Therapeutics, University of Turku, Turku, Finland; 2 Department of Information and Service Economy, Aalto University School of Economics, Helsinki, Finland; 3 Department of Information and Computer Science, Aalto University School of Science, Helsinki, Finland; State University of Rio de Janeiro, Biomedical Center, Institute of Biology, Brazil

## Abstract

**Aims:**

The antidiabetic drug metformin is currently used prior and during pregnancy for polycystic ovary syndrome, as well as during gestational diabetes mellitus. We investigated the effects of prenatal metformin exposure on the metabolic phenotype of the offspring during adulthood in mice.

**Methods:**

Metformin (300 mg/kg) or vehicle was administered orally to dams on regular diet from the embryonic day E0.5 to E17.5. Gene expression profiles in liver and brain were analysed from 4-day old offspring by microarray. Body weight development and several metabolic parameters of offspring were monitored both during regular diet (RD-phase) and high fat diet (HFD-phase). At the end of the study, two doses of metformin or vehicle were given acutely to mice at the age of 20 weeks, and Insig-1 and GLUT4 mRNA expressions in liver and fat tissue were analysed using qRT-PCR.

**Results:**

Metformin exposed fetuses were lighter at E18.5. There was no effect of metformin on the maternal body weight development or food intake. Metformin exposed offspring gained more body weight and mesenteric fat during the HFD-phase. The male offspring also had impaired glucose tolerance and elevated fasting glucose during the HFD-phase. Moreover, the expression of GLUT4 mRNA was down-regulated in epididymal fat in male offspring prenatally exposed to metformin. Based on the microarray and subsequent qRT-PCR analyses, the expression of Insig-1 was changed in the liver of neonatal mice exposed to metformin prenatally. Furthermore, metformin up-regulated the expression of Insig-1 later in development. Gene set enrichment analysis based on preliminary microarray data identified several differentially enriched pathways both in control and metformin exposed mice.

**Conclusions:**

The present study shows that prenatal metformin exposure causes long-term programming effects on the metabolic phenotype during high fat diet in mice. This should be taken into consideration when using metformin as a therapeutic agent during pregnancy.

## Introduction

Metformin is a biguanide class of antidiabetic agent and it is the most commonly prescribed oral drug for the treatment of patients with type 2 diabetes. The main pharmacological effect of metformin is decreased hepatic gluconeogenesis [1], [2]. Additionally, it has been shown to improve insulin sensitivity, lipid profile, endothelial function, and body weight control [2]. Thus metformin possesses a favourable spectrum of actions for the treatment of overweight, type 2 diabetes patients. Several studies have shown that metformin exerts its beneficial metabolic effects through AMP-activated protein kinase (AMPK) [3], [4]. AMPK is a cellular energy regulator that activates ATP-producing metabolic pathways [5],[6] and autophagy [7], [8] to normalise the energy balance of the cells.

In addition to the primary indication of metformin, it is increasingly used prior and during gestation for the treatment of polycystic ovary syndrome (PCOS). Furthermore, it is used to prevent or treat the complications arising from gestational diabetes mellitus (GDM) [9]–[16]. Metformin has been shown to reduce insulin resistance, lower ovarian androgen production, facilitate conception [17] and reduce first-trimester miscarriages [15] in PCOS patients. In addition to this it is also beneficial in preventing fetal macrosomy in pregnancies of GDM patients [14]. However, due to the challenges in study design, the knowledge of the long-term effects of prenatal metformin exposure is still scarce.

Barker's hypothesis of the connection between poor prenatal environment and increased disease susceptibility at adult age received attention over two decades ago [18]. The hypothesis has gained mechanistic support from further experimental studies and broadened to a significant field of research of developmental programming [19]–[22]. We hypothesised that prenatal metformin exposure might have long-term effects and here we show results which suggest that prenatal metformin exposure has a major impact on the metabolic phenotype of the offspring.

## Methods

### Ethics statement

Animal work was planned and performed according to the Finnish Act on the Use of Animals for Experimental Purposes and the study scheme was approved by the Finnish Animal Experiment Board (Permit ESAVI-2010-06188). During the studies, animals were monitored for any signs of morbidity and all efforts were made to minimise suffering.

### Animals

10–15 week-old C57/BL6NHsd mice (Harlan Laboratories, NL) were used for mating. Mice were housed on a 12 h∶12 h dark∶light cycle and fed either a regular diet (RD) containing 69% carbohydrates, 22% protein and 9% fat resulting in 3.6 kcal/g (diet CRM(E), SDS, UK) or high fat diet (HFD) containing 20% carbohydrates, 20% protein and 60% fat resulting in 5.2 kcal/g (diet D12492, Research Diets, NJ, USA) *ad libitum*. The lipid source of the high fat diet is lard and contains 32% saturated, 35.9% monounsaturated and 32% polyunsaturated fats. All percentages are expressed as w/w.

### Study Design

**Figure 1:**
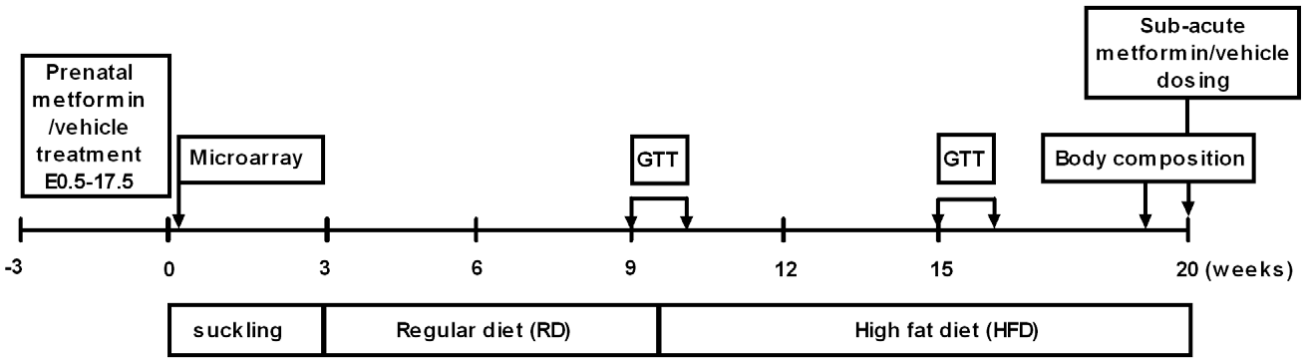
Schematic timeline of the study. Metformin was administered from E0.5 to E17.5 to dams on a regular diet. Week 0 denotes the birth. GTT = glucose tolerance test.

For mating, a male mouse was introduced to a female before the onset of the dark period and removed from the cage in the morning. The morning of the detection of a seminal plug in the female, was designated as embryonic day 0.5 (E0.5). Metformin (Sigma-Aldrich, St. Louis, Missouri, USA) (300 mg/kg) or vehicle (tap-water) was administered orally from E0.5 to E17.5 to the dams. Dams were fed RD during the pregnancy (see above) and the food intake was measured daily by collecting and weighing the pellets carefully from the grid and cage. If required, litter size was adjusted from 6 to 9 by introducing or separating pups between dams in the same prenatal treatment group. At the age of 3 weeks, offspring were weaned to RD (i.e. the RD-phase). The offspring continued on HFD from the age of 9–10 weeks until the end of the experiment (i.e. the HFD-phase). Body weight was measured once or twice *per* week. At the end of the study, the offspring of both prenatal groups were randomised to receive two doses of metformin (300 mg/kg p.o.) or vehicle 24 hours and one hour prior to killing. Terminal anesthesia was induced with a pre-made mixture of medetomidine∶ketamine∶saline in ratio of 2∶3∶5. The stock concentrations for medetomidine (Domitor®, Orion Pharma, Espoo, Finland) and ketamine (Ketalar®, Pfizer, Helsinki, Finland) were 1 mg/ml and 50 mg/ml, respectively (medetomidine 0.002 mg/g and ketamine 0.13 mg/g). Separate identical studies were performed in order to generate samples for the microarray study and the metformin concentration measurements (see below). Figure 1 represents a schematic drawing of the experimental model used in this study.

### Illumina microarray analysis

The samples for the Illumina microarray (Illumina Sentrix Mouse WG-6 v2 Expression Bead Chip, The Finnish Microarray and Sequencing Centre, Turku, Finland) were taken from 4-day old female and male offspring which had been exposed to metformin or vehicle during gestation (see the protocol for gestational treatment above). Neonatal mice were killed by decapitation. Livers (female and male mice) and brains (male mice) for RNA isolation were immersed in RNAlater reagent (Qiagen) and stored at −70°C. RNA was isolated using a RNAeasy Minikit (Qiagen) according to manufacturer's instructions with an additional DNase I digestion (Qiagen) step. For the array, 400 ng of total RNA was amplified with Illumina® TotalPrep™-96 RNA Amplification Kit (Applied Biosystems) and used for *in vitro* -transcription and biotinylation reactions. cRNA was hybridised to the Illumina Sentrix Mouse WG-6 v2 Expression Bead Chip -microarray chip which detects more than 45200 transcripts. Hybridisation was detected with Cyanine3-strepavidin. The chips were then scanned with Illumina Bead Array Reader. The quality of the total RNA and cRNA was validated with Agilent 2100 Bioanalyzer prior to the microarray (The Finnish Microarray and Sequencing Centre, Turku, Finland).

The microarray data was analysed using the R software package (http://www.r-project.org/). Probes with a detection P-value<0.05 in each comparison were included in the analysis. The data was normalised using quantile normalisation (beadarray package). For each comparison, differentially expressed genes were identified using Bioconductor limma package with moderated t-statistic with a false discovery rate (FDR)<0.05. Hierarchical clustering with Euclidean distance and complete linkage method was performed for the genes that were differentially expressed in at least one comparison.

### Gene set enrichment analysis (GSEA)

GSEA was performed with version 2.08 [23], [24] by using microarray expression values and comparing control and metformin groups both in females and males. Default parameters were used and permutations were performed using gene sets as the number of samples in each group was small. Expression values of the microarray analysis included 17122 features that could be collapsed into 12568 genes. Gene set databases BIOCARTA [25], KEGG [26], [27] and REACTOME [28] were used (downloaded from Board Institute at MIT). These gene sets databases consisted 92, 155 and 269 gene sets that met the analysis criteria (minimum 15 genes and maximum 500 genes in a gene set). Gene sets having P-value and FDR q-value<0.05 were considered enriched.

### Quantitative Real-Time PCR (qRT-PCR)

500 ng of total RNA (isolated as described above) was transcribed to cDNA with the DyNAmo cDNA synthesis kit (Thermo Scientific) using random hexamer primers. qRT-PCR was performed using the SYBR green (Kapa Biosystems, MA, USA) method in a ABI 7300 Real Time PCR system. The final concentration of forward and reverse primers in the reaction was 0.2 µM. The data was analysed according to the 2^−ΔΔCt^ method [29] using β-actin as an endogenous control gene. Primer sequences from 5′ to 3′ are shown in Table 1.

**Table 1:** The sequences of the qRT-PCR primers (from 5′ to 3′).

Gene (Accession)	*Forward primer*	*Reverse primer*
Insig-1 (NM_153526.5)	CAGCGGAATGTCACGCTCTT	AGGGATACAGTAAACCGACAACA
GLUT4 (NM_009204.2)	GACGGACACTCCATCTGTTG	GCCACGATGGAGACATAGC
β-actin (NM_007393.3)	TCCATCATGAAGTGTGACGT	GAGCAATGATCTTGATCTTCA

### Serum metformin

The quantitative analysis of metformin concentration in mouse serum was performed using a validated method consisting of a reversed phase liquid chromatography (Shimadzu Prominence HPLC instrument, Shimadzu Corporation, Kyoto, Japan) combined with mass spectrometric detection (AB Sciex API4000 triple quadrupole mass spectrometer, MA, USA). 180 µl of serum was mixed with 800 µl of a 40% (v/v) methanol solution containing 0.1% formic acid and 20 µl of an internal standard solution (Phenformin (Sigma) 20 µg/ml in water). The HPLC separation was carried out with a C18 reversed phase chromatography column (Ultrasophere C18 5 ODS 250 mm×4.6 (HiCrom, Berkshire, UK)). The triple quadrupole mass spectrometer was used in multiple reaction -monitoring mode. The chromatograms were analysed and processed using Analyst 1.4 software (AB Sciex).

### Body composition

Body composition was measured by quantitative nuclear magnetic resonance (NMR) scanning (EchoMRI-700, Echo Medical Systems) at the end of the HFD-phase. The mouse was restrained to a transparent cylinder tube and the measurement was performed twice. The percentage of fat, lean and total water mass was calculated relative to the body weight according to the methods provided by the manufacturer.

### White adipose tissue (WAT) depot and liver weights

After anaesthesia and decapitation of the mouse, inguinal, gonadal/epididymal, retroperitoneal and mesenteric fat depots and whole livers were excised and weighed. The collection of tissue samples was carried out during day (approximately from 9.00 to 15.00) and was assigned randomly so that both prenatal treatment groups were equally distributed over the time period.

### Glucose tolerance test (GTT)

GTTs for the offspring were performed at the age of 9–10 weeks (during RD) and 15–16 weeks (during HFD). Following 4 hours of fasting, the basal level of blood glucose was measured. Glucose (1 mg/g) was injected intraperitoneally (i.p.) and the blood glucose concentration was measured from a blood sample obtained from the tail vein 20, 40, 60 and 90 minutes after glucose injection (Precision Xceed, Abbot Diabetes Care Ltd, Witney, Oxon, UK).

### Serum lipids

Serum samples for the analysis of the lipid profile were obtained via cardiac puncture under anaesthesia. Circulating triglycerides and non-esterified fatty acids (NEFA) were quantified by Serum Triglyceride Determination Kit (Sigma-Aldrich, St. Louis, Missouri, USA) and NEFA Determination Kit (Wako Pure Chemical Industries, Osaka, Japan), respectively. Total cholesterol levels were quantified with Cayman Cholesterol Kit (Cayman Chemical, Michigan, USA).

### Statistical analyses

Two-way repeated-measures ANOVA (2-way RM-ANOVA) with a Bonferroni *post hoc* test was used for the analyses of body weight development, glucose tolerance, and fasting glucose. The body weight development was analysed separately during the RD- and HFD-phases of the offspring. In order to compare two groups, Student's t-test or Mann-Whitney test was used. A 2-way ANOVA was used when analysing the data from the prenatal groups randomised to receive sub-acute metformin or vehicle. All statistical analyses were performed with GraphPad Prism 5.0. The data is expressed as mean ± SEM. Results were considered statistically significant if P<0.05.

## Results

### Body weight development and food intake during gestation and prenatal weight of fetuses at E18.5 and serum metformin concentrations

**Figure 2:**
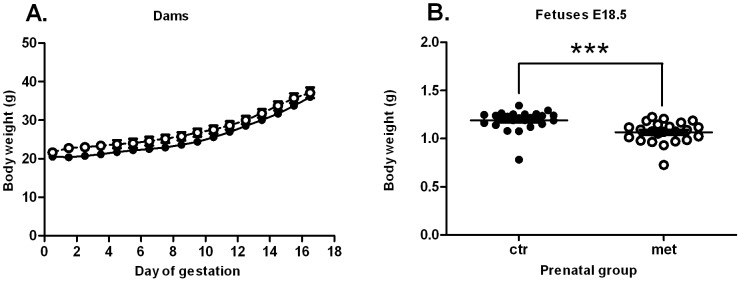
Body weight development during gestation and the weight of the fetuses at E18.5. Body weight development of the dams does not change due to metformin treatment (2A) (n = 5–7). Prenatal metformin exposure decreases body weight of the fetuses (2B) (n(litters) = 3, n(fetuses) = 23 for both groups). Black circles (•) = prenatal control, white circles (○) = prenatal metformin. Data expressed as mean ± SEM. ***P<0.0001 by Student's t-test.

Metformin treatment (300 mg/kg/day) during gestation did not change the body weight development (Figure 2A) or food intake (data not shown) of the dams to a significant degree. Nevertheless, metformin exposed fetuses had a significantly reduced body weight at E18.5 (Figure 2B).

Using a separate set of animals, the concentration of metformin in the serum of the dams and fetuses at E18.5 after 24–29 hours from the last administration showed that the dams were exposed to174±114 (n = 4) nmol/l and the fetuses to 130±21 nmol/l (n = 26) of drug. Metformin exposure was relatively similar in dams and fetuses indicating a good penetration through the placenta. There were no differences in the drug concentrations between sexes.

### Body weight development of the offspring during RD-phase and HFD-phase

**Figure 3:**
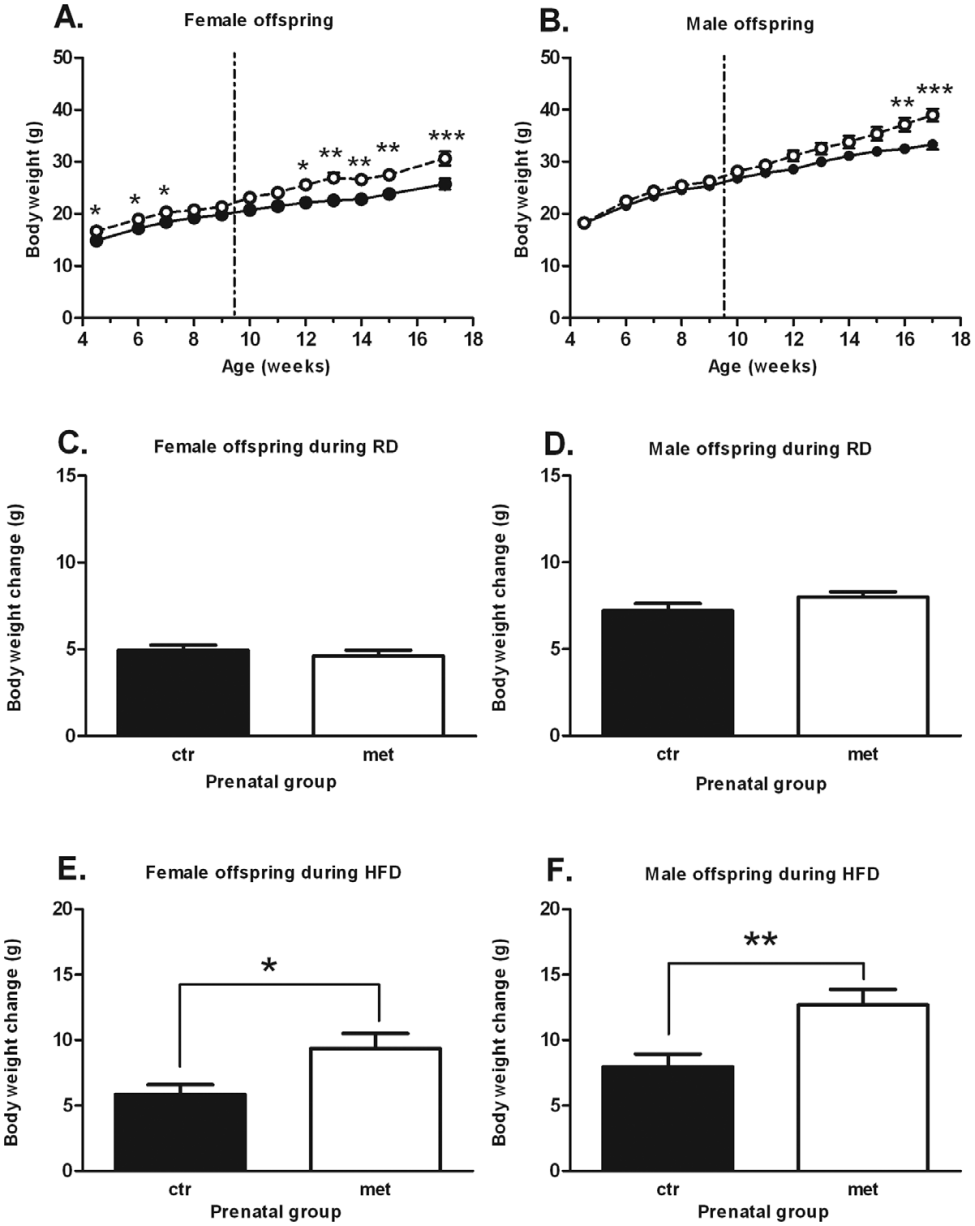
Body weight development of the offspring during RD and HFD. Body weight development of the female (A) and male offspring (B) (n = 13–18). The dotted vertical line indicates the diet change from RD to HFD. Black circles (•) = prenatal control, white circles (○) = prenatal metformin. Total body weight change during RD and HFD for female (C, E) offspring and male (D, F) offspring. Data expressed as mean ± SEM. *P<0.05, **P<0.001, ***P<0.0001 by Bonferroni *post hoc* tests for body weight development and Student's t-test for total body weight change.

The female offspring which were exposed to metformin were observed to be on a higher body weight curve during the RD-phase from 4.5 to 7 weeks of age (Figure 3A). No differences were noted in the body weight development of the male offspring during the RD-phase (Figure 3B). The body weight change during the RD-phase did not differ in either sex (Figure 3C and D). However, when the offspring were exposed to the HFD from 9-week of age onwards, both female and male offspring exposed to metformin gained significantly more body weight than the control group during the 8-week period (Figures 3A and B). Total body weight change during the HFD-phase was 5.9 g±0.7 g for the control group and 9.4 g±1.1 g for the metformin group (P = 0.0148) in the female offspring (Figure 3E) and 8.0 g±1 g for the control group and 12.7 g±1.2 g for the metformin group (P = 0.0043) in the male offspring (Figure 3F).

### Body composition of the offspring at the end of HFD-phase

**Figure 4:**
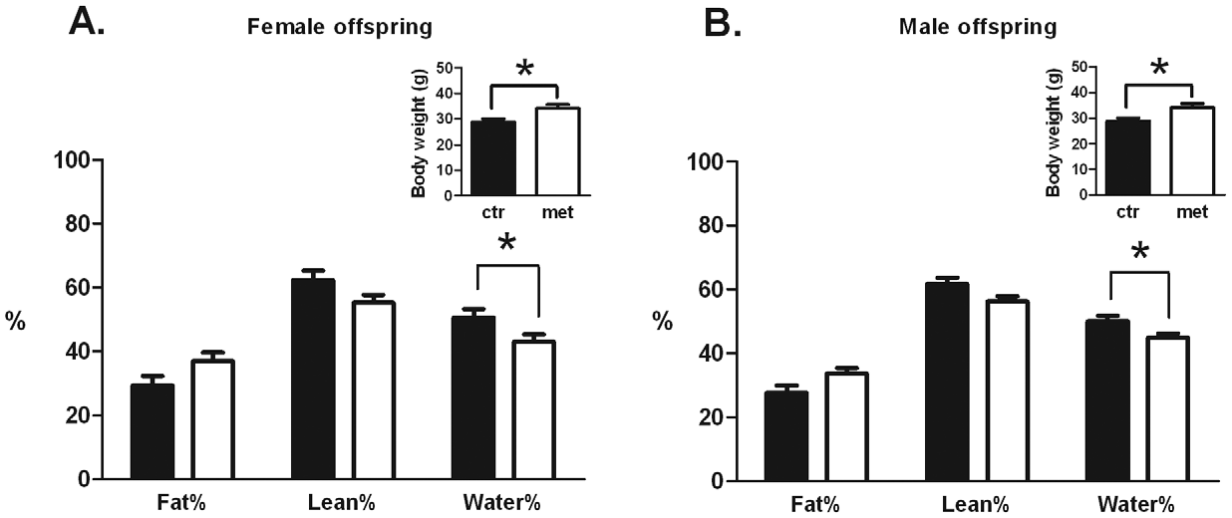
Body composition.

Body composition of the offspring was determined using NMR scanning at 19 weeks of age (Figure 4). Metformin exposed female offspring had a significantly (P = 0.0498) reduced water content. In addition to this, there was a tendency (P = 0.0777) towards reduced lean mass and a tendency (P = 0.1089) towards increased fat mass content (Figure 4A). A similar body composition profile of the metformin group in compared to the control group was measured in the male offspring ((P(water%) = 0.0404, P(lean%) = 0.0516, P(fat%) = 0.0561) (Figure 4B)).

**Figure 5:**
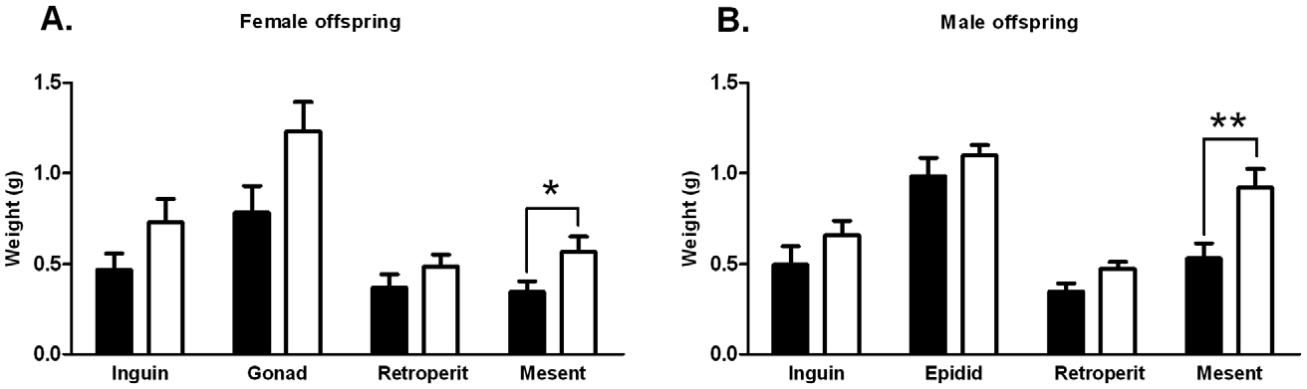
Weights of WAT depots. WAT depots were excised at the age of 20 weeks (n = 9–16). Black columns = prenatal control, white columns = prenatal metformin. Data expressed as mean ± SEM. *P<0.05, **P<0.001 by Student's t-test or Mann-Whitney test.

Four different white adipose tissue (WAT) depots (inguinal, gonadal/epididymal, retroperitoneal and mesenteric) were analysed by excising the required tissue at the age of 20 weeks (Figure 5). Metformin exposed female offspring had significantly (P = 0.0405) more mesenteric fat (0.34 g±0.06 g in the control group and 0.57 g±0.09 g in the metformin group) and a tendency (P = 0.0598) to an increased amount of gonadal fat ((0.78 g±0.15 g in the control group and 1.23 g±0.16 g in the metformin group) (Figure 5A)). Metformin exposed male offspring showed a significant (P = 0.0063) increase in mesenteric fat (0.53 g±0.08 g in the control group and 0.92 g±0.1 g in the metformin group) and a tendency (P = 0.0514) to a higher amount of retroperitoneal fat ((0.35 g±0.05 g in the control group and 0.47 g±0.04 g in the metformin group (Figure 5B)).

### The effects of the prenatal exposure to metformin on glucose tolerance

**Figure 6:**
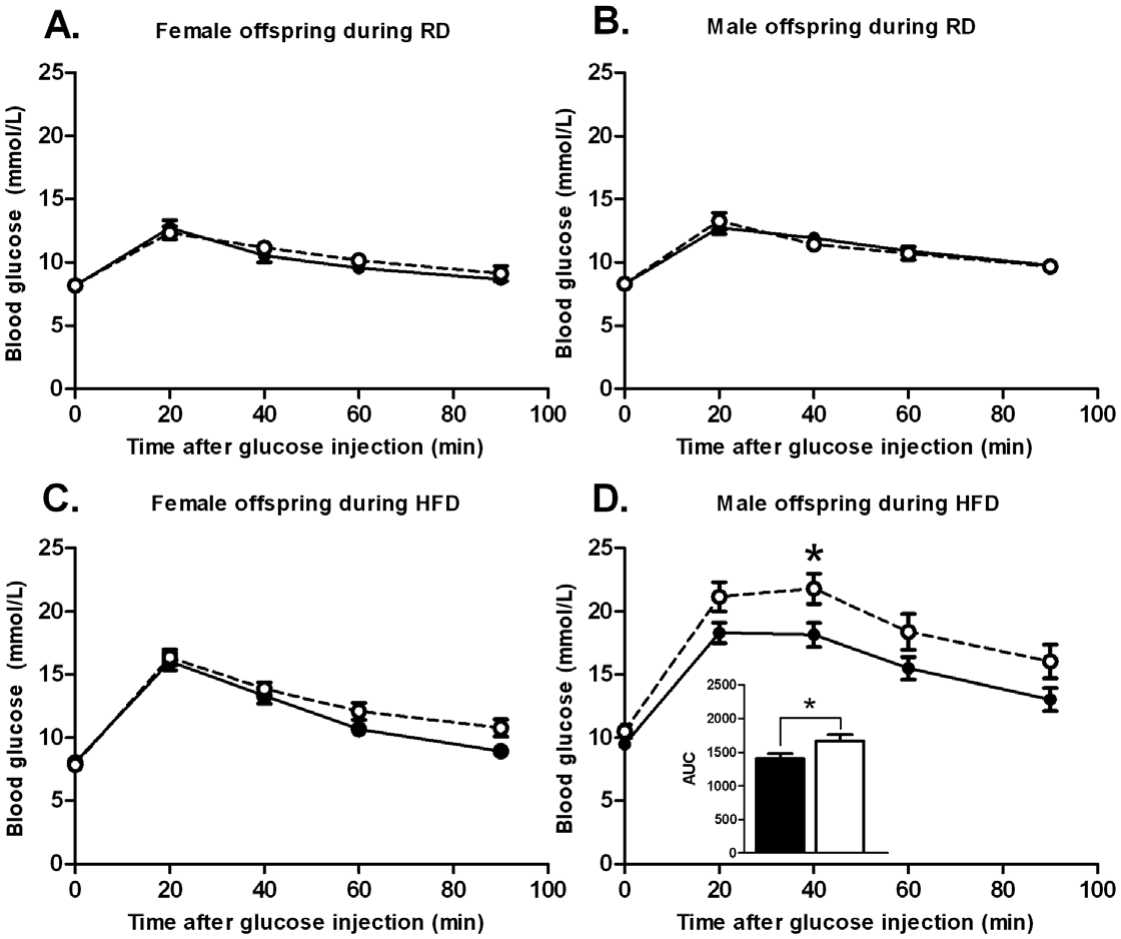
Prenatal metformin exposure alters glucose tolerance in the male offspring during HFD in adulthood. Glucose tolerance was tested during RD (A, B) (age = 9–10 weeks) and after 5–6 weeks of a HFD intervention (age = 15–16 weeks) (C, D) (n = 12–19) Black circles (•) = prenatal control, white circles (○) = prenatal metformin. Data expressed as mean ± SEM. *P<0.05 by Bonferroni *post hoc* tests.

**Figure 7:**
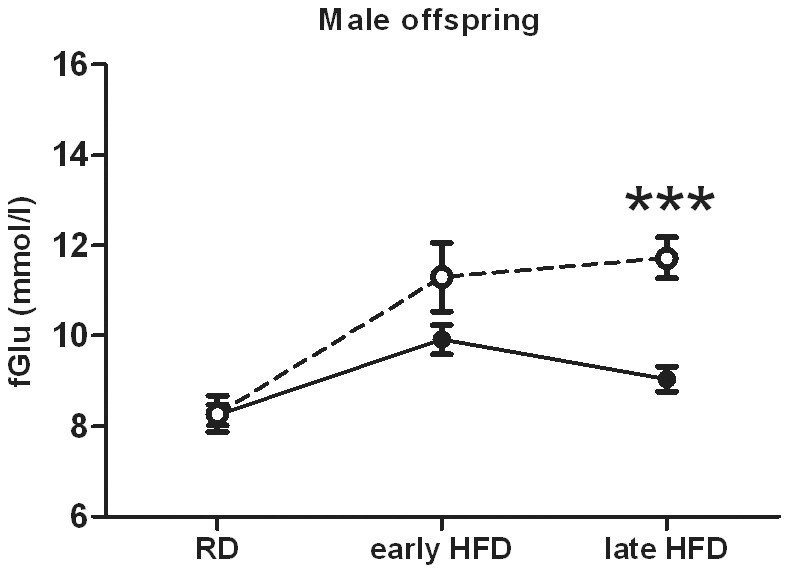
Prenatal metformin exposure leads to elevated fasting glucose during HFD in the male offspring. Fasting glucose was significantly elevated after a 10-week HFD intervention in metformin exposed male offspring. Time points: RD = 9–10 weeks, early HFD 16 weeks, late HFD = 19 weeks. Black circles (•) = prenatal control, white circles (○) = prenatal metformin (n = 12–16). Data expressed as mean ± SEM. ***P<0.0001 by Bonferroni *post hoc* tests.

Glucose tolerance of the offspring did not differ in the early development during the RD-phase (Figures 6A and B). Female offspring did not show differences in their glucose tolerance during the HFD-phase (Figure 6C). However, metformin exposed male offspring showed impaired glucose tolerance compared to the control group during the HFD-phase ((AUC, P = 0.0320; prenatal treatment effect P = 0.0298 by 2-way RM-ANOVA) (Figure 6D)). Moreover, consistent with the impaired glucose tolerance, fasting glucose was severely elevated in metformin exposed male offspring ((P<0.0 01 by Bonferroni *post hoc* test at a 10-week time point for the HFD-phase) (Figure 7)).

### The effects of prenatal metformin exposure on liver weight and lipid profile of the offspring during the HFD-phase

Both female and male offspring exposed to metformin had significantly (P = 0.0021 for females; P = 0.0062 for males) enlarged livers by weight compared to the control group at the study endpoint ((0.92 g±0.04 g in the control group and 1.17 g±0.06 g in the metformin group of female offspring; 1.42 g±0.06 in the control group and 1.77 g±0.1 g in the metformin group of male offspring (Table 2)). The analysis of the lipid profile demonstrated little changes between the groups. Metformin exposed female offspring had significantly (P = 0.0317) higher total cholesterol levels compared to the control group.

**Table 2:** Liver weight and lipid profile.

	*Females*			*Males*		
	*ctr*	*met*	*P-value*	*ctr*	*met*	*P-value*
Liver (g)	0.92±0.04	1.17±0.06	0.0021	1.42±0.06	1.77±0.1	0.0062
NEFA (mmol/l)	0.19±0.02	0.25±0.05	0.2857	0.23±0.02	0.22±0.007	0.852
Triglycerides (mg/ml)	0.28±0.05	0.23±0.02	0.7302	0.44±0.02	0.43±0.04	0.7771
Cholesterol (mmol/l)	1.85 ±0.24	3.01±0.35	0.0317	3.90±0.32	4.23±0.29	0.4638

Liver weights, serum NEFA, triglycerides and total cholesterol were analysed at 20 weeks of age (n = 4–8 for serum quantifications and n = 9–16 for liver weights) from control and metformin exposed offspring (prenatal group). Data expressed as mean ± SEM. P values by Student's t-test or Mann-Whitney test.

### Prenatal metformin exposure affects gene expression at neonatal age

**Figure 8:**
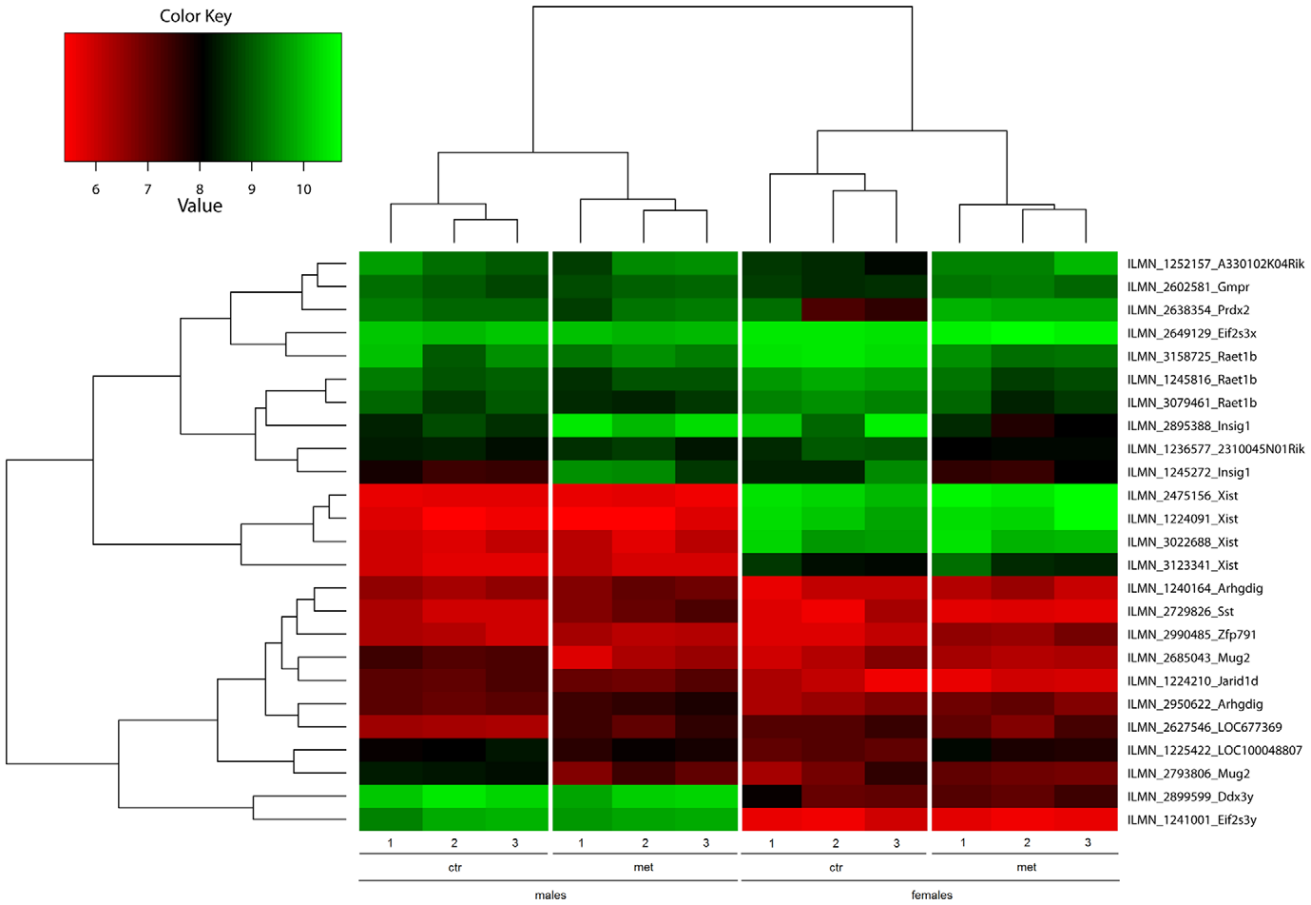
Hepatic gene expression profile in the neonatal offspring. Heatmap with hierarchical clustering of the hepatic gene expression profile of 4-day old offspring (n = 3 in each group).

Illumina microarray analysis of liver (female and male offspring) and brain (male offspring) gene expression was performed on 4-day old offspring which received metformin or vehicle during E0.5 - E17.5 (n = 3 for all groups). The expression of *Insig-1, LOC100048807, Prdx2, Raet1b, Zfp791, A330102K04Rik, Gmpr* and *2310045N01Rik* was altered according to the microarray data in the livers of the female offspring and *Mug2, LOC677369, Insig-1* and *Sst* in the livers of the male offspring. In the male brain, *LOC100041416* and *Zfp239* were changed in response to metformin exposure. Table 3 illustrates the significantly (P<0.05, adjusted P-value) differentially expressed genes (prenatal metformin vs. control). Figure 8 represents a heatmap of differentially expressed genes in the liver (analysis derived from metformin vs. control and male vs. female comparisons).

**Table 3:** Differentially expressed genes in the microarray in the liver (female and male offspring) and the brain (male offspring) following prenatal metformin exposure (n = 3).

*Symbol*	*Definition*	*RefSeqID*	*logFC*	*P-value (adjusted)*	*Probe*
***Liver (females)***					
*Insig-1*	Insulin induced gene-1	NM_153526.2	1.9	0.0143	ILMN_2895388
*LOC100048807*	PREDICTED: Hypothetical protein LOC100048807	XM_001471648.1	−0.8	0.0427	ILMN_1225422
*Prdx2*	Peroxiredoxin 2	NM_011563.2	−1.8	0.0428	ILMN_2638354
*Raet1b*	Retinoic acid early transcript beta	NM_009017.1	1.0	0.0428	ILMN_3158725
*Zfp791*	Zinc finger protein 791	NM_001037745.1	−0.8	0.0428	ILMN_2990485
*Raet1b*	Retinoic acid early transcript beta	NM_009017.1	0.8	0.0428	ILMN_1245816
*A330102K04Rik*	PREDICTED: RIKEN cDNA A330102K04 gene	XR_001572.1	−1.2	0.0428	ILMN_1252157
*Gmpr*	Guanosine monophosphate reductase	NM_025508.3	−0.7	0.0428	ILMN_2602581
*2310045N01Rik*	PREDICTED: RIKEN cDNA 2310045N01, transcript variant 10	XM_922052.2	0.7	0.0495	ILMN_1236577
***Liver (males)***					
*Mug2* *	Murinoglobulin 2	NM_008646.3	−1.1	0.0172	ILMN_2685043
*LOC677369*	PREDICTED: Similar to Alpha-2-macroglobulin	XR_005046.1	1.0	0.0172	ILMN_2627546
*Insig-1* *	Insulin induced gene-1	NM_153526.2	1.6	0.0241	ILMN_2895388
*Insig-1*	Insulin induced gene-1	NM_153526.4	1.6	0.0309	ILMN_1245272
*Sst*	Somatostatin	NM_009215.1	1.0	0.0504	ILMN_2729826
*Mug2*	Murinoglobulin 2	NM_008646.1	−1.2	0.0504	ILMN_2793806
***Brain (males)***					
*LOC100041416*	PREDICTED: Hypothetical protein LOC100041416	XM_001476334.1	−1.1	0.0002	ILMN_2738233
*Zfp239*	Zinc finger protein 239, transcript variant 1	NM_001001792.1	1.2	0.0052	ILMN_2467917

LogFC = binary logarithmic scale of the fold change in gene expression (metformin vs. control).

* = Significant signal with two separate probes.

**Figure 9:**
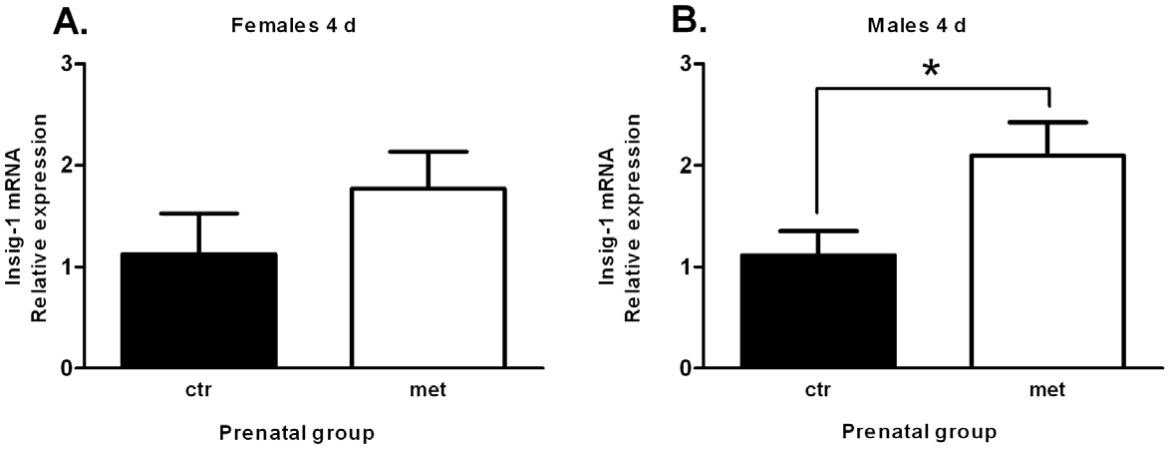
Hepatic Insig-1 expression is up-regulated by prenatal exposure to metformin. Hepatic Insig-1 expression in female and male offspring at day 4 (n = 3–7) (A, B).

As Insig-1has previously been connected to lipogenesis, adipogenesis and is induced in adipose tissue by a high fat diet [30], we chose to validate the microarray result concerning *Insig-1* gene expression by qRT-PCR. Insig-1 mRNA expression was significantly (P = 0.0487) increased in 4-day old male offspring (Figure 9B). The expression was also increased in females but without statistical significance (Figure 9A).

Gene set enrichment analyses (GSEA) revealed glyserolipid and lipoprotein metabolism pathways to be enriched in the metformin exposed offspring when compared to the control group in the liver of the male offspring. In the brain, respiratory electron transport was enriched in the male offspring exposed to metformin (see Supporting Information, Tables S1, S2 and S3).

### Insig-1 and GLUT4 mRNA expression at the end of the HFD-phase and after the sub-acute metformin exposure

**Figure 10:**
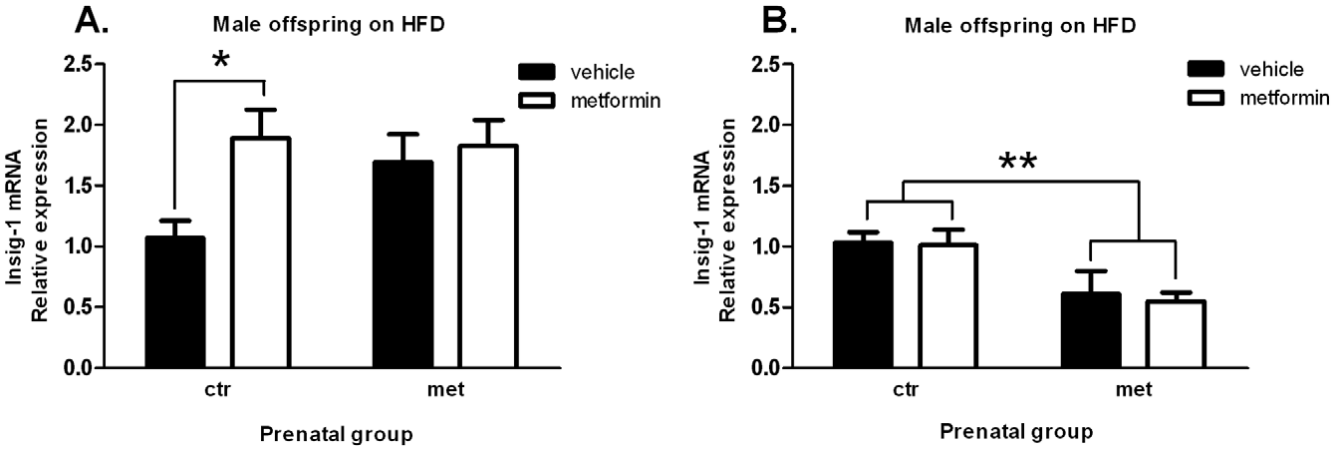
Insig-1 and GLUT4 mRNA expression at the end of the HFD-phase and after sub-acute metformin exposure. Insig-1 expression in liver (A) and GLUT4 expression in epididymal fat (B) at the study endpoint at the age of 20 weeks (n = 6–8). Columns grouped to the left = prenatal control, columns grouped to the right = prenatal metformin. Sub-acute vehicle = black, sub-acute metformin = white. Data expressed as mean ± SEM. *P<0.05 and **P<0.001 by 2-way ANOVA and Bonferroni *post hoc* tests.

In male offspring Insig-1 mRNA expression was significantly induced by a sub-acute metformin administration in the prenatal control group (Figure 10A) as compared to the loss of activation in the prenatal metformin group. Female offspring had no significant difference in response to the sub-acute metformin administration in regard to prenatal group (data not shown). In addition, at the study endpoint GLUT4 mRNA expression was significantly down-regulated in epididymal fat tissue in the male offspring which had been prenatally exposed to metformin (Figure 10B).

## Discussion

By studying the effects of prenatal metformin exposure in mice, we showed that exposure to metformin at fetal phase programs the metabolic phenotype of the offspring. In our study, we first showed that metformin treatment led to a slight but significant decrease in fetal weight at E18.5 without affecting maternal body weight development or food intake. In addition to this, we showed that prenatal metformin exposure caused long-term effects that became particularly evident during the HFD-phase at later development in both sexes: prenatal metformin exposure led to an increased body weight gain, a significant reduction in total body water content, a significant increase in mesenteric fat and liver weight at the end of the HFD-phase. Furthermore, metformin exposed male offspring showed impaired glucose tolerance and elevated fasting glucose during the high fat diet.

The expression of *Insig-1*, one of the hepatic genes up-regulated by metformin, which was observed in the microarray data, was detected to be up-regulated by a sub-acute metformin dose in later development in the male offspring from the prenatal control group. In the group exposed to prenatal metformin no such activation of this gene was observed. Finally, we demonstrated that GLUT4 mRNA expression in epididymal fat tissue was down-regulated in the metformin exposed male offspring. These principal findings strongly suggest that prenatal metformin exposure does have long-term effects and the altered gene expression profile in the liver during the early neonatal period might be a significant contributor to the phenotype observed in our study.

The dose (300 mg/kg) for metformin administration was selected based on previously published studies of the effects of metformin in rodents [31]–[34]. In order to calculate the human dose, an allometric body surface area method (BSA) [35] was used. 300 mg/kg for a mouse corresponds to 1.7 g of metformin to a 70 kg man. This correlates well to human studies, where the passage of metformin through placenta has previously been studied with the dose of 850 mg twice a day in pregnant women [36]. The concentrations measured from the maternal and umbilical vein serum, in the study of Vanky et al. [36], correlates to the concentrations measured in our study. The dose used was also evidently well tolerated since there were no observable side-effects on the dams (such as changes in the body weight development or food intake during pregnancy) and thus the obesity-prone phenotype of the offspring is most probably not a result of altered maternal behaviour or unspecific toxic effects of metformin. Metformin administration was stopped at embryonic day 17.5 to avoid excess stress prior to parturition.

Metformin exposed offspring had a phenotype which resembled that of offspring from maternal undernourishment models [37], [38]. In our study, the female offspring showed transient increase in body weight already on regular diet, however, the main difference in the body weight development between the prenatal groups occurred during the high fat diet. The food intake was not measured during the high fat diet, therefore, we cannot determine if the increased body weight originated from the excess intake of calories or decreased metabolic rate or a combination of these two factors.

Body composition measurements revealed that increased body weight was principally due to an increased amount of fat tissue in both sexes. By surgical excision, the most prominent weight change in the fat depots was observed in the mesenteric fat depot. We also observed that the liver weight was increased in metformin exposed offspring. The liver triglycerides were not measured in this study, however the increase in the liver weight coupled with increased amount of mesenteric fat strongly imply that the liver weight arises from increased fat accumulation in the liver [39].

The metabolic changes did not significantly concern blood lipid profile as at the end of our follow-up time for the offspring there were only minor changes observed. At this point the offspring had been on the high fat diet for 11 weeks and it might be that potential differences had already been blunted by the extensive time of high fat diet consumption. Moreover, as in other published studies related to prenatal programming [40]–[42] sexual dimorphism was also present in our model. Only the metformin exposed female offspring were shown to have higher cholesterol levels and the metformin exposed male offspring had impaired glucose metabolism.

Prenatal and early neonatal periods have been shown to be critical time periods for the development of energy balance and metabolic circuits [38], [43]. Predisposition to an atypical environment during this time window can result in sustained alterations in the phenotype [20], [37], [38], [40], [41], [44]–[48]. Therefore, we investigated the gene expression profile in the liver and brain from 4-day old offspring. There were significant changes in the hepatic gene expression profile according to the microarray analysis. The most consistent change in hepatic gene expression was seen with insulin induced gene-1 (Insig-1) which showed approximately a 3-fold up-regulation in the mRNA expression in response to prenatal metformin exposure in the microarray analysis. Subsequent qRT-PCR analyses confirmed significant increase in Insig-1 mRNA expression in the neonatal male offspring. Our result, showing the increase in Insig-1 mRNA expression in response to metformin is the first time this gene has been linked to the mode of action of metformin in the liver. Insig-1 is an endoplasmic reticulum (ER) protein involved in lipogenesis. It works through the inactivation of SREBPs [30], a transcription factor family for lipogenic enzymes, through binding to SREBP cleavage activating protein or SCAP and retaining the complex in the ER. Metformin on the other hand has previously been shown to decrease the expression of many lipogenic enzymes in liver [50]. Interestingly, metformin exposure has also been demonstrated to induce a fasting-like state in adult mice [51]. Moreover, Onken and Driscoll [52] have shown that metformin exposure mimics a dietary restriction-like state in *C.elegans* by activating insulin independent caloric restriction pathways through AMPK and LKB1. There has also been shown to be a direct connection of Insig-1 and AMPK expression in liver cells [53], although in our study, no difference in AMPK expression was detected either by microarray or qRT-PCR at the age of 4 days (data not shown). Gene set enrichment analyses of the microarray data from the liver showed pathways which are involved in glycerolipid and lipoprotein metabolism to be in enriched in the male offspring exposed to metformin. Moreover, several pathways involved in cell growth and proliferation were enriched in the control group when compared to the metformin exposed offspring. These findings are most probably connected to the direct effects of metformin as it is known that it affects lipid metabolism [3], [50] and as there is growing evidence of the anti-proliferative actions of metformin [54], [55]. As the fetus develops under the influence of this environment, these pathways might play a role in the later phenotype.

There was minimal differences in the gene expression within the neonatal brain following metformin exposure, only two genes with yet an undefined role in the metabolism were significantly changed. As metformin is a water-soluble agent, its access across the blood-brain barrier is limited and therefore this finding might be expected. There is also a possibility that the gene expression profile of the whole brain conceals subtle changes occurring in energy balance regulating regions such as in hypothalamic nuclei. However, gene set enrichment analyses revealed that processes connected to respiratory electron transport are enriched in the metformin exposed male offspring. Based on the current information we can only hypothesise the importance of the finding. The possible effect of metformin on the function of neuronal mitochondria and its contribution to central regulation of energy balance could be linked to the observed programming effect of metformin. It should be noted that the microarray analyses were based on a relatively small number of animals and therefore should be interpreted cautiously. Further studies are needed to elucidate the findings in this study.

The elevated fasting blood glucose of metformin exposed male offspring could not be explained by increased gluconeogenesis as analyzed by the mRNA expression of gluconeogenic marker genes GLUT2, G6pc, GCK, Pck1 and PPARγ1a (data not shown). However, what we did see was a down-regulation of GLUT4 mRNA expression in epididymal fat tissue. Previous studies have shown that intrauterine growth restriction can down-regulate the expression of GLUT4 by epigenetic mechanisms in the muscle of rats [56] and this potentially contributes to the development of metabolic disturbances in later development [57]. However, although the muscle is the primary tissue that responses to insulin, there are some indications that fat tissue expression of GLUT4 might actually be more important for the development of glucose intolerance [58] and decreased GLUT4 expression would be an indication of insulin-resistant state.

We investigated the effects of prenatal metformin exposure using pregnant inbred C57/BL6NHsd mice on a regular diet. Clinically there are two different type of pregnancy-related conditions for which metformin treatment is used for: polycystic ovary syndrome (PCOS) and gestational diabetes mellitus (GDM). PCOS affects approximately 10% of women of reproductive age and the main features include hyperandrogenemia, hirsutism, oligoanovulation and polycystic ovaries [59]. Furthermore, these women are also often characterised by insulin resistance and hyperinsulinemia. One of the clinical problems in PCOS arises from infertility and there are encouraging data for the use of metformin in PCOS, as demonstrated by Morin-Papunen [16]. As previously stated, the long-term effects of prenatal exposure to metformin are not known in humans. Interestingly, van Houten et al. (2012) [60] have recently published a mouse model mimicking human PCOS. However, because these mice were reported to be anestrous, this mouse model would not be optimal in our current study plan. Thus, our regular diet model has an opportunity to provide essential data before the corresponding long-term human data would be collected.

A clinical follow-up study of the offspring of GDM mothers treated with metformin showed, that when the offspring were 2 years old, there was no difference in the total fat content in the offspring exposed to metformin (alone or combined with insulin) compared to those exposed to insulin [61]. Interestingly, the distribution of fat was changed, with a hypothetical protective effect of an increase in subcutaneous fat in metformin/insulin group on the later accumulation of ectopic fat [61]. Our own preliminary data also shows that prenatal metformin exposure might protect weight gain during a high fat diet in the offspring when the dams have been on a high fat diet prior and during gestation (*unpublished data* Salomäki et al.). Based on different views on the use of metformin during pregnancy [36], [62], [63], we claim that it is justified to study the long-term effects of prenatal metformin exposure on the metabolic phenotype of the offspring in different nutritional and metabolic situations.

In summary, we observed a programming effect of prenatal metformin exposure that became evident during high fat diet at later stage of development. This was characterised by greater increases in body weight and mesenteric fat in both sexes. In addition to this, we observed impaired glucose tolerance in the male offspring. Importantly, the decreased expression of GLUT4 mRNA in epididymal fat at the end of the HFD-phase partly explained the metabolically disturbed phenotype of the male offspring which were prenatally exposed to metformin. Further experiments are required but we do suggest that the potential programming effect of metformin on metabolism should be taken into consideration in clinical situations.

## Supporting Information

Table S1 **Enriched hepatic pathways in the female offspring.** GSEA enriched pathways with P-value and FDR q-value threshold 0.05 are shown. Additionally, the normalised enrichment score (NES) is reported.
(DOCX)

Table S2 **Enriched hepatic pathways in the male offspring.** GSEA enriched pathways with P-value and FDR q-value threshold 0.05 are shown. Additionally, the normalised enrichment score (NES) is reported.
(DOCX)

Table S3 **Enriched central nervous system (CNS) pathways in the male offspring.** GSEA enriched pathways with P-value and FDR q-value threshold 0.05 are shown. Additionally, the normalised enrichment score (NES) is reported.
(DOCX)
