# Correction: Prenatal Metformin Exposure in Mice Programs the Metabolic Phenotype of the Offspring during a High Fat Diet at Adulthood

**DOI:** 10.1371/annotation/abe54d92-1f87-4826-a0a5-ba55005f99b4

**Published:** 2013-10-16

**Authors:** Henriikka Salomäki, Laura H. Vähätalo, Kirsti Laurila, Norma T. Jäppinen, Anna-Maija Penttinen, Liisa Ailanen, Juan Ilyasizadeh, Ullamari Pesonen, Markku Koulu

The y-axis for figure 10B is incorrectly labeled. The correct labeling is "GLUT4 mRNA". 

