# Supplementary material for: Prenatal Metformin Exposure in Mice Programs the Metabolic Phenotype of the Offspring during a High Fat Diet at Adulthood
Source: PLoS One. 2013 Feb 15;8(2):e56594. doi: 10.1371/journal.pone.0056594 (PMC3574083; doi:10.1371/journal.pone.0056594)
Supplement: Table S1 — Enriched hepatic pathways in the female offspring. GSEA enriched pathways with P-value and FDR q-value threshold 0.05 are shown. Additionally, the normalised enrichment score (NES) is reported. (DOCX) [file pone.0056594.s001.docx]

| **HEPATIC PATHWAYS**  **Females** |  | **NES** | **Nominal**  **P-value** | **FDR**  **q-value** |
| --- | --- | --- | --- | --- |
|  | ***KEGG PATHWAYS*** |  |  |  |
| ***Enriched in control group*** |  |  |  |  |
|  | KEGG CELL CYCLE | 2.210 | 0.000 | 0.000 |
|  | KEGG OOCYTE MEIOSIS | 1.737 | 0.000 | 0.001 |
|  | KEGG DNA REPLICATION | 2.005 | 0.000 | 0.002 |
|  | KEGG MISMATCH REPAIR | 1.947 | 0.000 | 0.004 |
|  | KEGG ECM RECEPTOR INTERACTION | 1.843 | 0.000 | 0.011 |
|  | KEGG NUCLEOTIDE EXCISION REPAIR | 1.847 | 0.000 | 0.013 |
|  | KEGG SPLICEOSOME | 1.776 | 0.000 | 0.024 |
|  | KEGG PROGESTERONE MEDIATED OOCYTE MATURATION | 1.730 | 0.000 | 0.036 |
|  |  |  |  |  |
|  | ***BIOCARTA PATHWAYS*** |  |  |  |
| ***Enriched in control group*** |  |  |  |  |
|  | BIOCARTA CELLCYCLE PATHWAY | 2.371 | 0.000 | 0.000 |
|  | BIOCARTA G2 PATHWAY | 2.272 | 0.000 | 0.000 |
|  | BIOCARTA MCM PATHWAY | 2.221 | 0.000 | 0.000 |
|  | BIOCARTA G1 PATHWAY | 1.957 | 0.000 | 0.005 |
|  | BIOCARTA RAC1 PATHWAY | 1.878 | 0.000 | 0.013 |
|  | BIOCARTA EDG1 PATHWAY | 1.855 | 0.001 | 0.015 |
|  | BIOCARTA TPO PATHWAY | 1.807 | 0.002 | 0.022 |
|  | BIOCARTA EIF4 PATHWAY | 1.766 | 0.007 | 0.029 |
|  | BIOCARTA HCMV PATHWAY | 1.751 | 0.009 | 0.029 |
|  | BIOCARTA P53HYPOXIA PATHWAY | 1.676 | 0.007 | 0.039 |
|  | BIOCARTA FCER1 PATHWAY | 1.711 | 0.004 | 0.040 |
|  | BIOCARTA BCR PATHWAY | 1.694 | 0.008 | 0.041 |
|  | BIOCARTA GH PATHWAY | 1.677 | 0.008 | 0.042 |
|  | BIOCARTA RACCYCD PATHWAY | 1.681 | 0.004 | 0.043 |
|  | BIOCARTA GCR PATHWAY | 1.695 | 0.007 | 0.044 |
|  | BIOCARTA ECM PATHWAY | 1.647 | 0.009 | 0.049 |
|  | ***REACTOME PATHWAYS*** |  |  |  |
| ***Enriched in control group*** |  |  |  |  |
|  | REACTOME CELL CYCLE MITOTIC | 1.933 | 0.000 | 0.001 |
|  | REACTOME MRNA SPLICING | 1.934 | 0.000 | 0.001 |
|  | REACTOME GLUCOSE TRANSPORT | 1.928 | 0.000 | 0.001 |
|  | REACTOME GLOBAL GENOMIC NER | 1.936 | 0.000 | 0.001 |
|  | REACTOME SNRNP ASSEMBLY | 1.893 | 0.000 | 0.002 |
|  | REACTOME E2F TRANSCRIPTIONAL TARGETS AT G1 S | 1.894 | 0.000 | 0.002 |
|  | REACTOME REV MEDIATED NUCLEAR EXPORT OF HIV1 RNA | 1.901 | 0.000 | 0.002 |
|  | REACTOME ELONGATION AND PROCESSING OF CAPPED TRANSCRIPTS | 1.897 | 0.000 | 0.002 |
|  | REACTOME FORMATION AND MATURATION OF MRNA TRANSCRIPT | 1.882 | 0.000 | 0.002 |
|  | REACTOME PLC BETA MEDIATED EVENTS | 1.877 | 0.000 | 0.002 |
|  | REACTOME METABOLISM OF RNA | 1.855 | 0.000 | 0.003 |
|  | REACTOME DOUBLE STRAND BREAK REPAIR | 1.852 | 0.002 | 0.003 |
|  | REACTOME G2 M TRANSITION | 1.822 | 0.000 | 0.004 |
|  | REACTOME RNA POLYMERASE II TRANSCRIPTION | 1.792 | 0.000 | 0.007 |
|  | REACTOME TELOMERE MAINTENANCE | 1.788 | 0.001 | 0.007 |
|  | REACTOME SMOOTH MUSCLE CONTRACTION | 1.768 | 0.001 | 0.009 |
|  | REACTOME PLATELET DEGRANULATION | 1.765 | 0.000 | 0.009 |
|  | REACTOME MITOTIC M M G1 PHASES | 1.769 | 0.000 | 0.009 |
|  | REACTOME S PHASE | 1.753 | 0.000 | 0.009 |
|  | REACTOME CELL CYCLE CHECKPOINTS | 1.755 | 0.000 | 0.010 |
|  | REACTOME HIV LIFE CYCLE | 1.745 | 0.000 | 0.010 |
|  | REACTOME DUAL INCISION REACTION IN GG NER | 1.730 | 0.001 | 0.012 |
|  | REACTOME PLATELET ACTIVATION | 1.726 | 0.000 | 0.012 |
|  | REACTOME GENE EXPRESSION | 1.722 | 0.000 | 0.013 |
|  | REACTOME CENTROSOME MATURATION | 1.714 | 0.000 | 0.014 |
|  | REACTOME DEADENYLATION OF MRNA | 1.703 | 0.009 | 0.015 |
|  | REACTOME RNA POLYMERASE I PROMOTER OPENING | 1.696 | 0.001 | 0.016 |
|  | REACTOME SYNTHESIS OF DNA | 1.691 | 0.000 | 0.016 |
|  | REACTOME RNA POLYMERASE I CHAIN ELONGATION | 1.680 | 0.005 | 0.016 |
|  | REACTOME DNA REPAIR | 1.692 | 0.000 | 0.016 |
|  | REACTOME G1 S TRANSITION | 1.680 | 0.000 | 0.016 |
|  | REACTOME LATE PHASE OF HIV LIFE CYCLE | 1.681 | 0.000 | 0.016 |
|  | REACTOME LOSS OF NLP FROM MITOTIC CENTROSOMES | 1.682 | 0.000 | 0.017 |
|  | REACTOME NUCLEOTIDE EXCISION REPAIR | 1.685 | 0.001 | 0.017 |
|  | REACTOME TRANSCRIPTION | 1.683 | 0.000 | 0.017 |
|  | REACTOME TRANSLATION INITIATION COMPLEX FORMATION | 1.646 | 0.002 | 0.022 |
|  | REACTOME MUSCLE CONTRACTION | 1.647 | 0.003 | 0.022 |
|  | REACTOME TRANSCRIPTION COUPLED NER | 1.650 | 0.004 | 0.022 |
|  | REACTOME GENERIC TRANSCRIPTION PATHWAY | 1.648 | 0.011 | 0.022 |
|  | REACTOME DNA REPLICATION PRE INITIATION | 1.643 | 0.000 | 0.022 |
|  | REACTOME RNA POLYMERASE I PROMOTER CLEARANCE | 1.639 | 0.001 | 0.023 |
|  | REACTOME HIV INFECTION | 1.627 | 0.000 | 0.025 |
|  | REACTOME FORMATION OF PLATELET PLUG | 1.614 | 0.000 | 0.028 |
|  | REACTOME ADP SIGNALLING THROUGH P2Y PURINOCEPTOR 1 | 1.616 | 0.009 | 0.028 |
|  | REACTOME MRNA SPLICING MINOR PATHWAY | 1.601 | 0.010 | 0.032 |
|  | REACTOME PYRIMIDINE METABOLISM | 1.588 | 0.016 | 0.035 |
|  | REACTOME FURTHER PLATELET RELEASATE | 1.586 | 0.012 | 0.035 |
|  | REACTOME METABOLISM OF MRNA | 1.582 | 0.007 | 0.035 |
|  | REACTOME OPIOID SIGNALLING | 1.583 | 0.003 | 0.036 |
|  | REACTOME TRANSLATION | 1.579 | 0.001 | 0.036 |
|  | REACTOME GTP HYDROLYSIS AND JOINING OF THE 60S RIBOSOMAL SUBUNIT | 1.571 | 0.002 | 0.038 |
|  | REACTOME NCAM1 INTERACTIONS | 1.572 | 0.007 | 0.038 |
|  | REACTOME INFLUENZA LIFE CYCLE | 1.558 | 0.001 | 0.042 |
|  | REACTOME APOPTOTIC EXECUTION PHASE | 1.550 | 0.009 | 0.043 |
|  | REACTOME FORMATION OF THE EARLY ELONGATION COMPLEX | 1.551 | 0.017 | 0.044 |
|  | REACTOME FORMATION OF THE TERNARY COMPLEX AND SUBSEQUENTLY THE 43S COMPLEX | 1.544 | 0.009 | 0.045 |
|  | REACTOME SIGNAL AMPLIFICATION | 1.545 | 0.021 | 0.045 |
|  | REACTOME REGULATION OF INSULIN SECRETION BY ACETYLCHOLINE | 1.534 | 0.033 | 0.048 |
|  | REACTOME HIV1 TRANSCRIPTION INITIATION | 1.535 | 0.018 | 0.048 |
|  | REACTOME RNA POLYMERASE I. III AND MITOCHONDRIAL TRANSCRIPTION | 1.530 | 0.001 | 0.049 |
