# Supplementary material for: Prenatal Metformin Exposure in Mice Programs the Metabolic Phenotype of the Offspring during a High Fat Diet at Adulthood
Source: PLoS One. 2013 Feb 15;8(2):e56594. doi: 10.1371/journal.pone.0056594 (PMC3574083; doi:10.1371/journal.pone.0056594)
Supplement: Table S2 — Enriched hepatic pathways in the male offspring. GSEA enriched pathways with P-value and FDR q-value threshold 0.05 are shown. Additionally, the normalised enrichment score (NES) is reported. (DOCX) [file pone.0056594.s002.docx]

| **HEPATIC PATHWAYS**  **Males** |  | **NES** | **Nominal**  **P-value** | **FDR**  **q-value** |
| --- | --- | --- | --- | --- |
|  | ***KEGG PATHWAYS*** |  |  |  |
| ***Enriched in control group*** |  |  |  |  |
|  | KEGG NON SMALL CELL LUNG CANCER | 1.687 | 0.001 | 0.033 |
|  | KEGG PHOSPHATIDYLINOSITOL SIGNALING SYSTEM | 1.695 | 0.000 | 0.043 |
|  | KEGG DNA REPLICATION | 1.742 | 0.001 | 0.044 |
| ***Enriched in metformin group*** |  |  |  |  |
|  | KEGG GLYCEROLIPID METABOLISM | -1.510 | 0.000 | 0.060 |
|  | ***BIOCARTA PATHWAYS*** |  |  |  |
| ***Enriched in control group*** | BIOCARTA MCM PATHWAY | 1.830 | 0.001 | 0.010 |
|  | BIOCARTA TPO PATHWAY | 1.836 | 0.000 | 0.015 |
|  | BIOCARTA EGF PATHWAY | 1.725 | 0.002 | 0.030 |
|  | BIOCARTA G1 PATHWAY | 1.683 | 0.000 | 0.032 |
|  | BIOCARTA HCMV PATHWAY | 1.693 | 0.003 | 0.035 |
|  | BIOCARTA HER2 PATHWAY | 1.637 | 0.002 | 0.035 |
|  | BIOCARTA PDGF PATHWAY | 1.668 | 0.003 | 0.036 |
|  | BIOCARTA GH PATHWAY | 1.651 | 0.001 | 0.039 |
|  | BIOCARTA CASPASE PATHWAY | 1.639 | 0.000 | 0.040 |
|  | BIOCARTA CELLCYCLE PATHWAY | 1.616 | 0.008 | 0.041 |
|  | BIOCARTA GLEEVEC PATHWAY | 1.600 | 0.009 | 0.042 |
|  | BIOCARTA FCER1 PATHWAY | 1.602 | 0.000 | 0.045 |
|  | BIOCARTA IGF1 PATHWAY | 1.586 | 0.005 | 0.047 |
|  | ***REACTOME PATHWAYS*** |  |  |  |
| ***Enriched in control group*** |  |  |  |  |
|  | REACTOME ACTIVATION OF THE PRE REPLICATIVE COMPLEX | 1.977 | 0.000 | 0.002 |
|  | REACTOME DNA STRAND ELONGATION | 1.907 | 0.000 | 0.004 |
|  | REACTOME EXTENSION OF TELOMERES | 1.790 | 0.000 | 0.014 |
|  | REACTOME TELOMERE MAINTENANCE | 1.754 | 0.000 | 0.016 |
|  | REACTOME G2 M CHECKPOINTS | 1.762 | 0.000 | 0.018 |
|  | REACTOME ACTIVATION OF ATR IN RESPONSE TO REPLICATION STRESS | 1.720 | 0.000 | 0.023 |
|  | REACTOME COLLAGEN MEDIATED ACTIVATION CASCADE | 1.703 | 0.000 | 0.027 |
|  | REACTOME LAGGING STRAND SYNTHESIS | 1.679 | 0.001 | 0.029 |
|  | REACTOME E2F MEDIATED REGULATION OF DNA REPLICATION | 1.688 | 0.002 | 0.030 |
| ***Enriched in metformin group*** |  |  |  |  |
|  | REACTOME LIPOPROTEIN METABOLISM | -2.160 | 0.000 | 0.000 |
