# Supplementary material for: Prenatal Metformin Exposure in Mice Programs the Metabolic Phenotype of the Offspring during a High Fat Diet at Adulthood
Source: PLoS One. 2013 Feb 15;8(2):e56594. doi: 10.1371/journal.pone.0056594 (PMC3574083; doi:10.1371/journal.pone.0056594)
Supplement: Table S3 — Enriched central nervous system (CNS) pathways in the male offspring. GSEA enriched pathways with P-value and FDR q-value threshold 0.05 are shown. Additionally, the normalised enrichment score (NES) is reported. (DOCX) [file pone.0056594.s003.docx]

| **CNS PATHWAYS**  **Males** |  | **NES** | **Nominal**  **P-value** | **FDR**  **q-value** |
| --- | --- | --- | --- | --- |
|  | ***REACTOME PATHWAYS*** |  |  |  |
| ***Enriched in metformin group*** |  |  |  |  |
|  | REACTOME RESPIRATORY ELECTRON TRANSPORT | -2.010 | 0.000 | 0.023 |
